# Heterogeneous Cu–Fe oxide catalysts for preferential CO oxidation (PROX) in H_2_-rich process streams†

**DOI:** 10.1039/d0ra06969h

**Published:** 2020-09-30

**Authors:** Venkata D. B. C. Dasireddy, Krish Bharuth-Ram, Darko Hanzel, Blaž Likozar

**Affiliations:** Department of Catalysis and Chemical Reaction Engineering, National Institute of Chemistry Slovenia Hajdrihova 19 SI-1001 Ljubljana Slovenia venkata.dasireddy@ki.si; Physics Department, Durban University of Technology Durban 4000 South Africa; Jozef Stefan Institute Jamova 39 Ljubljana Slovenia

## Abstract

The influence of Fe loading in Cu–Fe phases and its effect on carbon monoxide (CO) oxidation in H_2_-rich reactant streams were investigated with the catalyst material phases characterized by Field Emission Scanning Electron Microscopy (FESEM), X-ray diffraction (XRD) studies and Mössbauer Spectroscopy (MS). There was no change in the oxidation state of the Fe ions with copper or iron loading. The catalytic activity was examined in the feed consisting of H_2_, H_2_O and CO_2_ for the preferential CO oxidation (PROX) process. These catalysts showed an optimized performance in converting CO in WGS streams in the temperature range of 80–200 °C. In addition to the formation of the CuFe_2_O_4_ phase, the Fe and Cu were found to be incorporated into a Cu–Fe supersaturated solid solution which improved CO oxidation activity, with carbon dioxide and water produced selectively with high catalytic activity in depleted hydrogen streams. Relatively high conversion of CO was obtained with high Fe metal loading. In addition to their catalytic efficiency, the employed heterogeneous catalysts are inexpensive to produce and do not contain any critical raw materials such as platinum group metals.

## Abstract 2

Fe loading in Cu–Fe phases and its effect on carbon monoxide oxidation in H_2_-rich reactant streams were investigated with the catalyst material phases characterized by Field Emission Scanning Electron Microscopy, X-ray diffraction studies and Mössbauer Spectroscopy.

## Graphical abstract

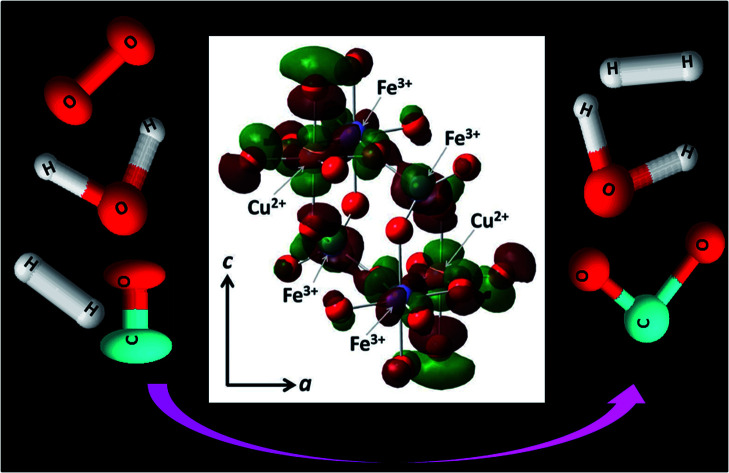

## 1 Introduction

The production of clean hydrogen for hydrogen-fuelled polymer electrolyte membrane fuel cells (H_2_-PEM) is gaining importance in recent days. The usage of these fuel cells has significantly lowered the carbon oxide emissions and increased renewable energy usage efficacy.^1^ There is still a challenge in the use of fuel cells for various mobile applications thus, vast research is concentrating on overcoming the difficulties with the supply and storage of clean hydrogen.^2^ The hydrogen for H_2_-PEM is produced in a fuel processing unit by partially oxidising or reforming the liquid fuels like liquefied petroleum gas (LPG), methanol or gasoline, which is further processed by a water–gas shift catalyst to produce a high quantity of hydrogen.^3^ The hydrogen-rich feed after the water–gas shift processing which is supplied to H_2_-PEM contains carbon residuals *i.e.* 1 mol% CO, which can poison the platinum anode catalyst used in H_2_-PEM cells, which further decreases the efficiency of the H_2_-PEM cell.^4^ Various methods were employed to decrease the CO from the H_2_ rich feed gas and among them, the preferential oxidation of CO (CO PROX) has been reported as the effective one to reduce the CO concentration to the minimum (>10 ppm), with a minimal loss of hydrogen.^5^ During the preferential oxidation of CO, the oxidation of hydrogen competes with CO oxidation which leads to a decrease of H_2_-PEM cell efficiency. Due to this, an active and selective catalyst is needed for the removal of CO from the H_2_ rich feed gas. A proper material for CO PROX reaction should have high activity, selectivity and stability from 80 to 220 °C.^6^

In a supported catalyst system, phase-specific mixed metal oxides have recently attracted great interest for use as catalyst and catalyst supports,^7,8^ since these materials give rise to well dispersed and stable metal particles on the surface of the support materials and consequently improved catalytic performance. In this regard iron(III) oxide catalysts, which have catalytic characteristics similar to other redox supports with oxygen storage capacities like ceria,^9^ zirconia^10^ and titania,^11^ but are considerably cheaper to produce, have attracted particular interest as catalysts for the preferential oxidation of carbon monoxide (CO PROX).

Iron oxides supported precious metal catalysts have been proven to be very effective for low-temperature CO oxidation. However, the high cost and limited availability restrict their use. In searches for an alternative, recent studies have found that Fe^3+^ had a promotional effect on the activity of Cu–Ce catalysts used for CO oxidation.^12^ The Cu-based oxide catalysts have good catalytic performances for CO-PROX, which coupled with their low fabrication costs make them an efficient alternative to the precious metal catalysts. In the copper–iron composite system, the primary active phase is CuFe_2_O_4_. However, the role of CuFe_2_O_4_ on CO oxidation has not been explored yet, and it is still a challenge to develop a Cu–Fe composite material for the desired enhancement of catalytic performance. To prepare the effective catalysts for the CO-PROX reaction, one could apply solution routes and a low-temperature treatment for combining the Fe and Cu oxides in one sample. In this study, Cu–Fe/Al_2_O_3_ and Cu and Fe loaded CuFe_2_O_4_, were synthesized (with a 5 wt% of Cu and 5 wt% of Fe loading) and the role of Fe species on the active phase and in the activity of the catalyst were investigated using Mössbauer spectroscopy and X-ray diffraction.

## 2 Experimental

Three sets of catalysts, Cu–Fe/Al_2_O_3_ and Cu and Fe loaded CuFe_2_O_4_, were synthesized as described below.

### (i) Cu–Fe/Al_2_O_3_ catalyst

A co-impregnation method is used for the preparation of the bimetallic catalyst containing 5 wt% of Cu and 5 wt% of Fe supported on Al_2_O_3_. The calculated quantities of metal nitrates (analytical grade, Sigma-Aldrich) were dissolved in sufficient quantities of deionized water. This solution was added drop-wise to Al_2_O_3_ powder which was suspended in 100 mL of deionized water which is under steady stirring (300 rpm). This mixture was aged for 5 h at 70 °C. The resulting paste was dried overnight at 90 °C and then calcined under a continuous airflow (100 mL min^−1^) at 300 °C for 4 h.

### (ii) CuFe_2_O_4_ bulk catalyst

CuFe_2_O_4_ bulk catalyst powders were prepared using wet chemical synthesis method. Appropriate stoichiometric quantities of copper and iron nitrate precursors required to make 3 g of the catalyst were added to 50 mL of distilled water. The solution was heated to dryness over a magnetic plate at 95 °C. The resulting brown solid was treated at 150 °C to allow the complete decomposition of nitrates and then calcined in a furnace at 500 °C for 4 h. The powder was removed from the furnace and reground in a mortar five times to facilitate the reaction.

### (iii) Cu/CuFe_2_O_4_ and Fe/CuFe_2_O_4_ catalysts

The catalysts consisted of 5 wt% Cu and 5 wt% Fe supported on CuFe_2_O_4_ were prepared using the traditional wet impregnation method. The calculated quantities of metal nitrates (analytical grade, Sigma-Aldrich) metal nitrates (Sigma-Aldrich) were first dissolved in sufficient quantities of deionized water. This metal nitrate solution was added drop-wise to prepared CuFe_2_O_4_ bulk catalyst powders which were suspended in 100 mL of deionized water under steady stirring (300 rpm). A paste was yielded after stirring this mixture for 5 h at 70 °C and it was dried overnight at 90 °C and then calcined under a continuous airflow (100 mL min^−1^) at 300 °C for 4 h. The catalysts obtained were denoted as Cu/CuFe_2_O_4_ and Fe/CuFe_2_O_4_ catalysts.

## 3 Characterisation

Physisorption analyses (Brunauer–Emmett–Teller (BET) surface area and pore volume studies) were carried out by degassing the catalysts under the N_2_ flow for 4 h at 200 °C using the Micrometrics FlowPrep 060. The degassed samples were analysed in the Micrometrics ASAP 2020 multi-point BET surface area analyser. The measured specific surface areas for the samples in crystallite forms were converted to equivalent particle size according to the following equation:^13–15^
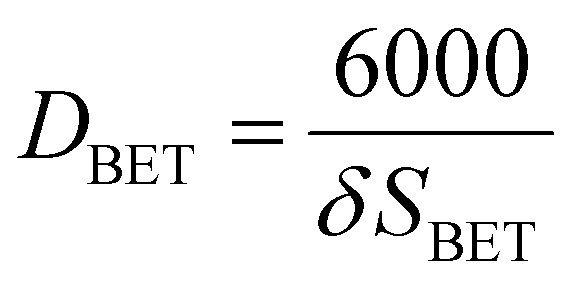
*D*_BET_ is the particle diameter in nm, *δ* is the material density in g cm^−3^, and *S*_BET_ is the surface area in m^2^ g^−1^.

Powder X-ray diffraction (XRD) studies were conducted using the PANalytical X'Pert Pro. The scans from 10 to 90° were carried out using the CuKα radiation source the wavelength of 1.5406 nm. Particle size, morphology and elemental mapping, performed by energy-dispersive X-ray spectroscopy (EDXS) analysis, were further investigated using the Cs-corrected scanning transmission electron microscope (SEM) (JEOL, JEM-ARM200CF), equipped with JEOL EDXS system. ^57^Fe transmission Mössbauer Spectroscopy measurements made at room temperature (RT) with a 
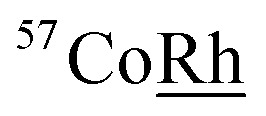
 source.

Temperature programmed chemisorption of H_2_, O_2_, CO, CO_2_ and NH_3_ were performed using the Micromeritics 2920 Autochem II Chemisorption Analyser in the method illustrated in ref. 16. Prior to chemisorption analysis, a temperature-programmed reduction (TPR) was carried out on catalyst samples using the same instrument. Prior to the reduction of a sample in TPR, catalysts were degassing by heating under the stream of argon (30 mL min^−1^) at 400 °C for 30 min, and consequently cooling back to 80 °C. Thereafter, 4.9 mol% H_2_ in Ar was used as the reducing agent at the flow rate of 30 mL min^−1^. Samples were analysed from the 80 °C to 950 °C using the ramp rate of 10 °C min^−1^. Following reduction, the stream of helium (30 mL min^−1^) was used for pre-treatment under at 350 °C for 60 min and consequently cooling back to 80 °C. A selected gas (4.9 mol% H_2_ in Ar/5 mol% O_2_ in Ar/10 mol% CO in He/10 mol% CO_2_ in He/9.8 mol% NH_3_ in He) was then passed over the catalysts at the flow rate of 30 mL min^−1^ for 60 min. Excess gas was consequently removed by purging with helium for 30 min (30 mL min^−1^). The temperature was then again raised gradually to 600 °C by ramping at 10 °C min^−1^ under the flow of helium and the desorption data of H_2_, O_2_, CO, CO_2_ and NH_3_ was recorded separately. The dispersion of the metals on was calculated using CO chemisorption data, assuming the metal/CO chemisorption ratio of 1.^17,18^

## 4 Catalytic testing

CO PROX catalytic experiments using the synthesized materials were carried out in a reactor described in.^16^ Before the reactions, each catalyst was pre-treated *in situ* in the flow of He (50 mL min^−1^) at 400 °C for 1 h. Reactions were performed in the temperature range from 40 to 220 °C, using the steps of 20 °C. The catalytic tests were carried with a gas hourly space velocity (GHSV) of 60 000 h^−1^ using a feed consisted of 1 vol% CO, 1 vol% O_2_, 60 vol% H_2_ and He as the balance. 10 mol% H_2_O and CO_2_ either/each compound was added in the feedstock to examine the catalyst activity and stability. Outlet gases, together with CO, O_2_, H_2_O, CO_2_ and H_2_ were analysed by online quadrupole mass spectrometry (MS). The signals of MS were calibrated using the mixtures with different mole fractions of CO, O_2_, H_2_O, CO_2_ and H_2_ that is, to determine the mole composition of the gases in the outflow. All catalytic tests were carried out in duplicate and the values, obtained for CO conversion, exhibited the standard deviation below 2% with a carbon balance ranging between 99–101%. Conversions and CO_2_ selectivity were calculated from the mole fraction of products in the exit stream according to the following equations.^19^

1 CO conversion (%) = *X*_CO_ = (*n*_CO,in_ − *n*_CO,out_)/*n*_CO,in_

2 O_2_ conversion (%) = *X*_O_2__ = (*n*_O_2_,in_ − *n*_O_2_,out_)/*n*_O_2_,in_

3 H_2_ conversion (%) = *X*_H_2__ = (2(*n*_O_2_,in_ − *n*_O_2_,out_) − (*n*_CO,in_ − *n*_CO,out_))/*n*_H_2_,in_

4 CO_2_ selectivity (%) = *S*_CO_2__ = (*n*_CO,in_ − *n*_CO,out_)/(2(*n*_O_2_,in_ − *n*_O_2_,out_))

The apparent activity for CO oxidation was applied to compare the performance of catalysts. The activity towards the reactions of CO was expressed as the amount of the CO converted per mass of a catalyst per second, which was calculated by the following equation.

5 CO oxidation activity (kmol_CO_ s^−1^ kg_cat._^−1^) = *A*_CO_ = (*p Q*_in_*x*_CO,in_*X*_CO_)/(RTW)

## 5 Results and discussion

The structural properties of the prepared Cu–Fe based catalysts are shown in Table 1. It can be observed that supporting of metal oxide on the Al_2_O_3_ resulted in an apparent decrease of the surface area and pore volume which could be due to the blocking of some micropores and mesopores of the Al_2_O_3_ supports by Cu and Fe particles. This decrease in the surface area and pore volume could also be attributed to the high dispersion of metal oxides. A similar trend is observed for Cu/CuFe_2_O_4_ and Fe/CuFe_2_O_4_ catalysts. The surface oxygen groups present on the surface of CuFe_2_O_4_ support serve as specific anchoring sites for the supporting of metal oxide, which further result in the high dispersion on the surface of the catalyst. These surface oxygen-containing groups, including hydroxyl moieties, also increase the hydrophilicity of CuFe_2_O_4_ catalyst surface. In general, most of these metal oxides don't form nanoparticles, while they are anchored to the surface of the catalyst and they are highly dispersed on the support as a monolayer. Conversely, the calcination under air, which is performed after the impregnation can lead to the aggregation of metal oxide particles (anchored to the CuFe_2_O_4_ surface).

**Table 1:** Selected structural properties of prepared catalysts

Catalyst	Surface area (m^2^ g^−1^)	Pore volume (cm^3^ g^−1^)	Metal dispersion^a^ (%)	Particle size^b^ (nm)	Cu^c^ (wt%)	Fe^c^ (wt%)
γ-Al_2_O_3_	243	0.82	—	—	—	—
CuFe_2_O_4_	52	0.17	18.1	48	11	30
Cu–Fe/Al_2_O_3_	112	0.37	35.1	22	4.8	4.9
Cu/CuFe_2_O_4_	85	0.28	39.7	35	26	24
Fe/CuFe_2_O_4_	61	0.20	27.3	39	14	36

a Measured from N_2_O chemisorption.

b Particle size from BET method.

c From EDX analysis.

**Fig. 1:**
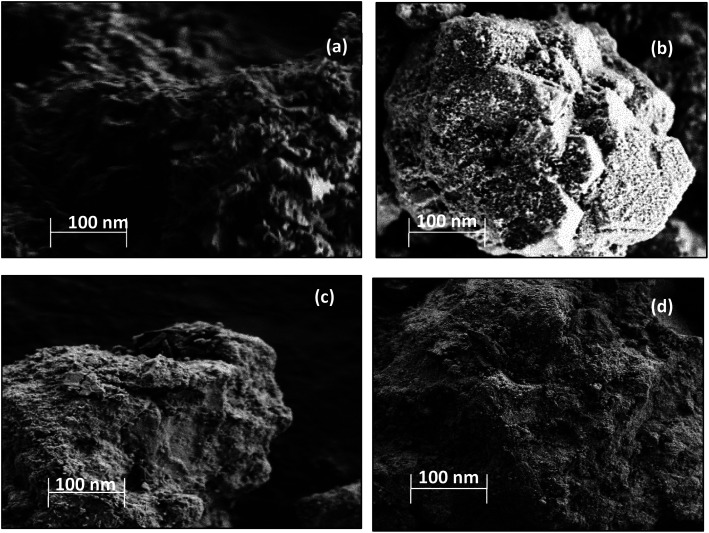
SEM scans of (a) CuFe_2_O_4_, (b) Cu–Fe/Al_2_O_3_, (c) Cu/CuFe_2_O_4_ and (d) Fe/CuFe_2_O_4_ catalysts.

The N_2_ adsorption and desorption isotherms obtained from physisorption analysis for the all the catalysts could be classified as type IV, which is typical for mesoporous materials.^20,21^ All the catalysts exhibited the hysteresis loop of type H1, indicating well-defined cylinder-like pore channels and uniform sphere agglomerates.^22^ The N_2_ adsorption–desorption isotherms along with pore size distributions showed an insignificant change over this catalyst (ESI, Fig. S1†), which indicates that the deposition of metal oxide on the surface of CuFe_2_O_4_ may introduce the defects without disturbing the pore structure. The surface morphology of the catalyst samples was studied using SEM scans, which are shown in Fig. 1.

The Cu–Fe/Al_2_O_3_ catalyst showed a circularly shaped particle, with evidence of agglomeration as well as nodular individual particles are seen in the structure (Fig. 1). In the CuFe_2_O_4_ based catalysts, the majority of the particles have rough surface morphology with Cu and Fe well dispersed on the surface.

**Fig. 2:**
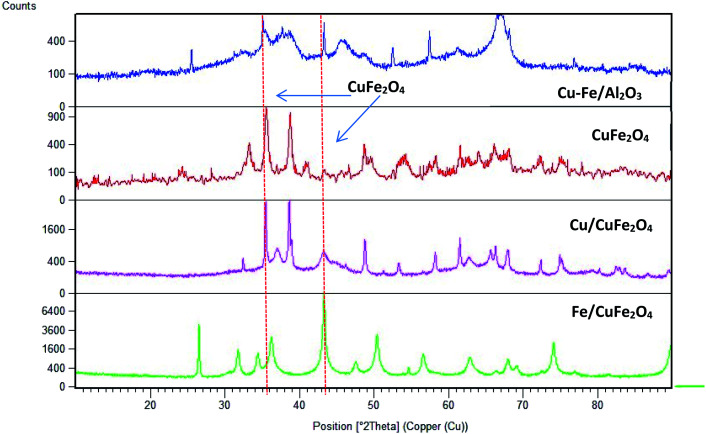
Powder X-ray diffraction scans of the prepared catalysts (CuFe_2_O_4_ phase is shown by dashed lines).

Fig. 2 displays the XRD patterns of the prepared catalysts. The main peak at a 2*θ* of 35.6° (002) and 38.5° (111) with a d-spacing of 2.52 Å and 2.33 Å are ascribed to the CuO monocrystalline phase (JCPDS, no. 48-1548). For bare CuFe_2_O_4_ and Cu–Fe/Al_2_O_3_ catalysts, the diffraction peaks were observed at 2*θ* values of 33.8°, 35.6°, 36.8° and 48.9° which are the characteristic peaks of CuFe_2_O_4_ phase (JCPDS no. 34-0425). This showed that copper may combine with iron to form Cu–Fe solid solution.^23^ In addition to this phase, there is the presence of iron oxide in the form of α-Fe_2_O_3_ (JCPDS no. 84-0307) as inferred from the diffraction peaks at a 2*θ* of 35.6°, 37.2° and 43.2°.

**Fig. 3:**
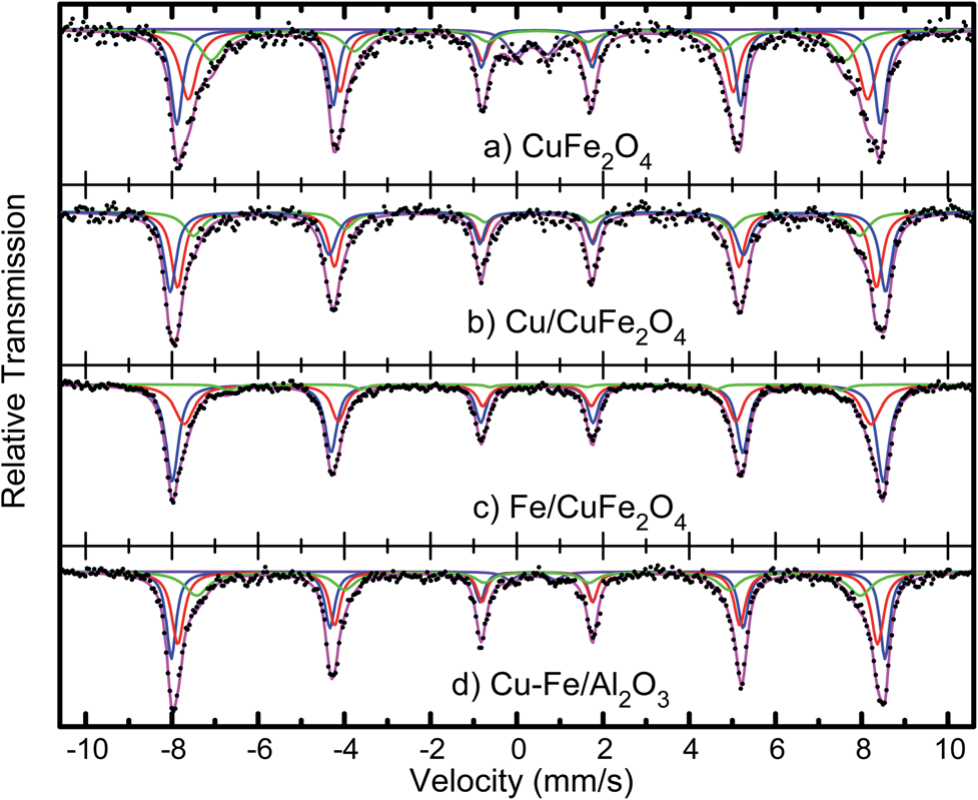
Mössbauer spectra of the CuFe_2_O_4_ catalysts, before and after Cu and Fe loading, and of Cu–Fe loaded Al_2_O_3_.

The average crystallite sizes in the Fe/Cu loaded catalysts, determined by using Scherrer equation from the linewidth of the line at 2*θ* = 36.8° were estimated to be in the range of 10–20 nm. In the presence of 5 wt% of Cu the CuFe_2_O_4_ catalyst showed only the reflections of monoclinic CuO structure (36.8°), and their diffraction intensity increases with the CuO content (Fig. 2), indicating the excessive CuO species in Cu–Fe systems. On the other hand, with a 5 wt% of Fe loading, a decrease in the crystallinity of the catalyst is observed. The Mossbauer spectra of CuFe_2_O_4_ catalyst and the Fe and Cu loaded CuFe_2_O_4_ and Cu–Fe loaded Al_2_O_3_ catalysts measured at room temperature are shown in Fig. 3.

The spectra were fitted with the analysis code RECOIL.^24,25^ Comparison of the spectrum of the CuFe_2_O_4_ sample with previous studies^26–28^ indicates that the minimum crystallites size in these samples is 30 nm. Further loading of Cu and Fe, followed by calcination at 400 °C, as described above, appears to reduce the size distribution and leads to better-defined components in the Mössbauer spectra. The spectra of the Cu and Fe loaded catalysts were fitted with three well-defined sextets belonging to the tetrahedral and octahedral sites of Fe^3+^ ions and confirm that the synthesised materials possess the inverse spinel structure. In case of an ideal inverse spinel structure, Cu^2+^ goes to one of the octahedral sites, one Fe^3+^ goes to the other octahedral site, and the other Fe^3+^ goes to the tetrahedral. Most probably due to some off-stoichiometry or defect structure, there is a mixture of normal and inverse spinel phases.

This ratio of the sextets depends on the excess iron or copper content in the catalyst. This indicates that copper combines with iron to form Cu–Fe supersaturated solid solution within the ferrite structure.

The measured values of isomer shift, quadrupole splitting and magnetic hyperfine field extracted from fits to the spectra are given in Table 2. The isomer shift values confirm that the Fe ions are in the 3+ state. No change in the oxidation state of iron ions is observed with an increase in copper or iron loading. Nor is there any systematic change in the isomer shift values of all the systems, implying that the s-electron density around the probe Fe nuclei is not much altered with the copper or iron addition.

**Table 2:** Mössbauer parameters, isomer shift (*δ*), electric quadrupole shift (*ε*), the hyperfine magnetic field and areal fraction, determined from the spectra shown in Fig. 3. The isomer shifts are expressed relative to α-Fe at room temperature

Sample	Component	*δ* (mm s^−1^)	*ε* (mm s^−1^)	H (kOe)	*Γ*/2 (mm s^−1^)	*f* (%)
CuFe_2_O_4_	Sx1	0.37(1)	−0.09(1)	506(1)	0.14	35(2)
	Sx2	0.36(1)	−0.10(1)	489(2)	0.18	37(4)
	Sx3	0.38(4)	−0.11(4)	456(4)	0.29	22(2)
	D1	0.34(6)	Δ*E*: 0.8(1)	—	0.25	6(2)
Cu/CuFe_2_O_4_	Sx1	0.36(1)	−0.10(1)	514(1)	0.14	40(2)
	Sx2	0.35(1)	−0.11(1)	503(2)	0.15	41(3)
	Sx3	0.36(4)	−0.13(4)	479(5)	0.20	19(5)
Fe/CuFe_2_O_4_	Sx1	0.36(1)	−0.11(1)	511(1)	0.15	59(2)
	Sx2	0.36(1)	−0.11(1)	494(2)	0.17	37(4)
	Sx3	0.46(5)	−0.05(5)	441(4)	0.15	4(2)
Cu–Fe/Al_2_O_3_	Sx1	0.36(1)	−0.10(1)	513(1)	0.13	36(2)
	Sx2	0.36(1)	−0.11(1)	503(2)	0.14	38(3)
	Sx3	0.37(3)	−0.10(3)	477(5)	0.25	24(5)
	D1	0.37(9)	Δ*E*: 1.1(2)	—	0.20	2(1)

**Fig. 4:**
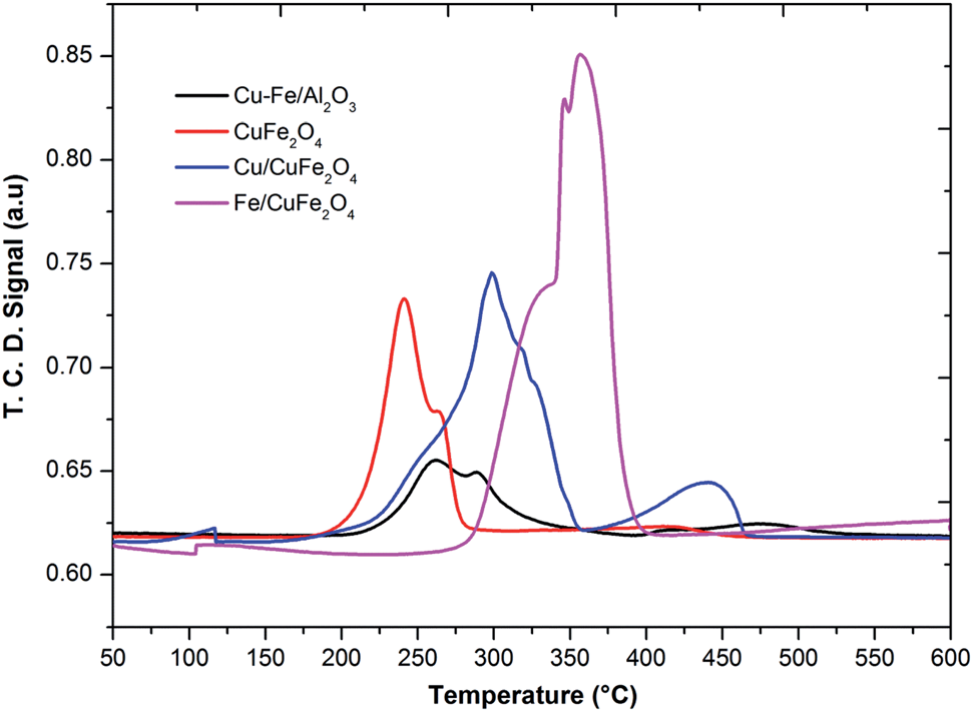
TPR profile of the prepared catalysts.

The TPR profile of prepared catalysts was shown in Fig. 4. In the H_2_-TPR profiles of catalysts, it can be seen there are two reduction peaks at low-temperature peak (below 250 °C) and a high-temperature peak (above 250 °C). For Cu– Fe/Al_2_O_3_ catalyst, these two peaks could be attributed to the reduction of highly dispersed and agglomerated CuO, respectively. The first peak (230 °C) could be assigned to the reduction of copper, which is from a monocrystalline phase of copper as shown in XRD. Moreover, it was considered that the advent of easily reducible oxide species at low temperature played a key role in shifting the TPR peaks of the cobalt and iron to lower temperature. In the TPR profile of the CuFe_2_O_4_ catalyst, one main peak with one shoulder peak appeared at the low-temperature region, corresponding to the reduction of CuO to Cu, Fe_2_O_3_ to Fe_3_O_4_ and the overlapped CuFe_2_O_4_ to Cu and Fe_2_O_3_ respectively.^23^

The reduction of Fe/CuFe_2_O_4_ occurred at a much higher temperature compared to CuFe_2_O_4_ and the broad peak detected at the high-temperature region for Fe/CuFe_2_O_4_ was attributed to the reduction of Fe_2_O_3_.^29^ The broad peak observed for the reduction of Fe can be attributed to the amorphous and well-dispersed iron oxide on the surface of CuFe_2_O_4_, which is also evidenced in the XRD results. In literature, it was reported that the Cu^2+^ and Fe^3+^ species in the mixed metal oxides were much more difficult to be reduced than that in plain CuO or Fe_2_O_3_.^23^ In comparison to the reduction peaks of monometallic copper, it could be found that the reduction of copper in the presence of iron and cobalt occurred at higher temperatures. This could be due to the strong interaction of copper with Fe or Co to support. It could be considered that the larger crystallite size may decrease in the density of metal on the surface strengthen the interaction of metals-support,^30^ making the Cu reduction of more difficult in the case of Fe/CuFe_2_O_4_ and Cu/CuFe_2_O_4_ catalysts.

CO-TPD was used for the determination and quantification of CO species present at the surface of the metal oxides.^31–33^ The amount of CO desorbed from supported catalysts is higher with compared to the bare CuFe_2_O_4_ catalyst. This could be due to the synergistic effect between the Cu/Fe which increases the metal active sites on the surface of the bare CuFe_2_O_4_ catalyst. With compared to the chemisorption of H_2_, the chemisorbed amount of CO and CO_2_ showed a lower amount for all the catalysts. The ratio between chemisorption capacity of H_2_ : CO and H_2_ : O_2_ is lower for Cu–Fe/Al_2_O_3_ catalyst with compared to all other catalysts which indicate that this catalyst might show a good activity towards the CO oxidation with compared to H_2_ oxidation (Table 2). The latter is very well discussed in the literature^34,35^ with the generation of an active site occurring when an oxide is deposited onto another to in turn form a surface-phase oxide. On the respective surface of the oxides of low oxidation state metals, metal-to-cation bond exhibits a highly ionic character, and thus, when this surface is crisp, the unsaturated metal cations can act as active sites. The strength of these surface active sites depends on the ionic character of the metal-to-cation oxygen bond, the ratio between the charge of the cation and its ionic radius, as well as its coordination.^36^ In the bimetallic mixed oxides, a simpler situation is represented by the case, in which only two types of cations are present. When the two have differed in oxidation states and electronegativity (Fe and Cu), those of one of the two components often dominate the global active site characteristics for chemisorption.^37,38^

**Fig. 5:**
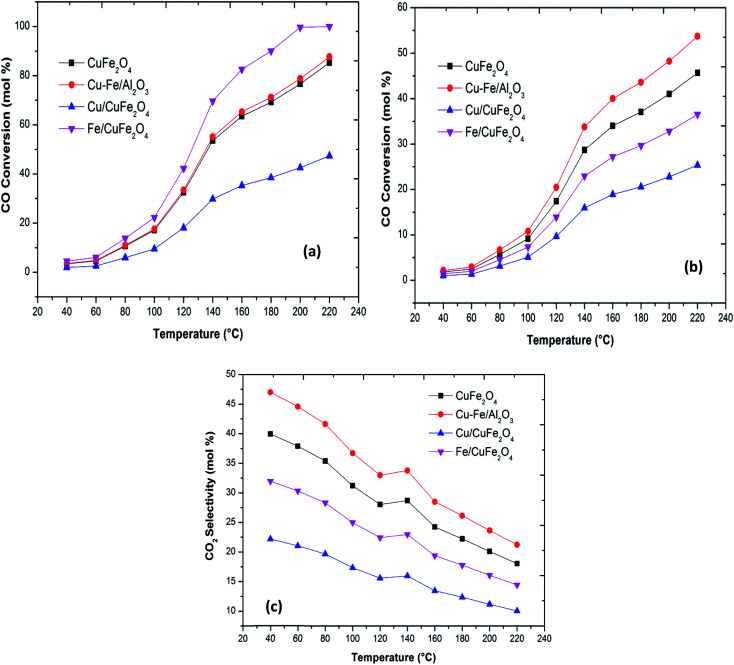
(a) CO oxidation (b) CO conversion in the presence of H_2_ and (c) CO_2_ selectivity in the presence of H_2_ with a reaction temperature of the Cu–Fe based catalysts.

Plots of CO oxidation and CO conversion and CO_2_ selectivity in the presence of H_2_ as functions of reaction temperature for the different catalysts are presented in Fig. 5. The Fe/CuFe_2_O_4_ catalyst performed best in CO conversion, achieving 100% conversion to CO_2_ at 200 °C. The least active was the Cu/CuFe_2_O_4_ catalyst, attributable to two likely causes: (a) incomplete reduction of Fe in the catalytic testing range, and/or (b) more severe agglomeration of Cu on the surface of CuFe_2_O_4_ (as reflected by the SEM and XRD scans).

The bare CuFe_2_O_4_ catalyst showed a very high CO conversion compared to the excess copper loaded catalyst. Thus, over the present catalysts, the activity shown by Cu–Fe metal oxide-based catalysts can be mainly attributed to the presence of dispersed iron oxides on the surface of CuFe_2_O_4_. The activities, thus, compare as follows at *T*_50_ (the temperatures where 50% CO conversion is achieved): Fe/CuFe_2_O_4_ (120 °C) > CuFe_2_O_4_/Al_2_O_3_ (140 °C) > CuFe_2_O_4_ (140 °C) > Cu/CuFe_2_O_4_. These results indicate that the introduction of Fe into CuFe_2_O_4_ catalysts can promote the oxidation of CO. The enhanced catalytic activity may be related to an increased availability of CO-free catalyst surface sites due to the incorporation of Fe on Cu sites.^11,12^ Our results of the enhanced activity in CO oxidation due to Fe loading has been previously observed by several researchers in the case of CeO_2_, Co_3_O_4_ and Mn_3_O_4_ (Table 3).^7,8^

**Table 3:** Particulate properties of the prepared catalysts

Catalyst	H_2_ chemisorbed (μmol H_2_ g^−1^)	O_2_ chemisorbed (μmol O_2_ g^−1^)	CO chemisorbed (μmol CO g^−1^)	CO_2_ chemisorbed (μmol CO_2_ g^−1^)
CuFe_2_O_4_	0.18	0.22	0.12	0.18
Cu–Fe/Al_2_O_3_	0.47	0.38	0.23	0.34
Cu/CuFe_2_O_4_	0.32	0.31	0.18	0.21
Fe/CuFe_2_O_4_	0.34	0.27	0.14	0.28

The PROX process involves two competitive reactions, the oxidation of carbon monoxide and the oxidation of hydrogen.^10,39^ The CO conversion on all prepared catalysts is significantly decreased in the presence of H_2_. The trend of CO_2_ formation decreases gradually from 45% to 25% with an increase in the temperature for Cu–CuFe_2_O_4_. The other catalysts show similar behaviour. The CuFe_2_O_4_ catalyst supported monometallic Cu or Fe catalysts show lower CO-PROX performance under identical conditions the reverse water gas shift reaction is not observed over these catalysts, in agreement with the literature.^8,40,41^ The negative effect of Cu or Fe for CO-PROX may therefore be attributed to the formation of the hydroxyl group, which can selectively oxidise hydrogen to water,^7,42,43^ the generation of an excess of –OH oxidising species bringing about a drop in CO_2_ selectivity, and hence leading to lower CO-PROX performance. The high selectivity towards CO_2_ can be attributed finely dispersed CuFe_2_O_4_ phase on Al_2_O_3_ as well as to the synergistic effect between Cu and Fe in CuFe_2_O_4_ phase.

**Fig. 6:**
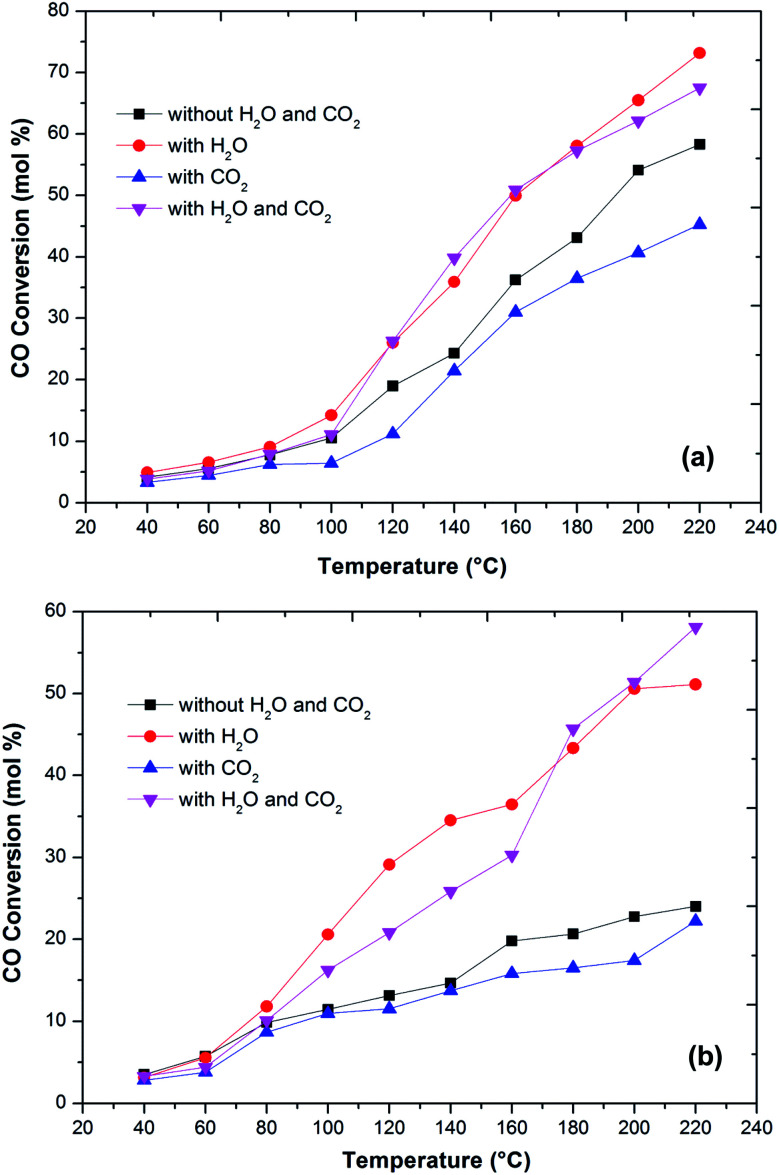
Variation of CO conversion over Cu–Fe/Al_2_O_3_ (a) and Cu/CuFe_2_O_4_ (b) catalyst with reaction temperature at different conditions (1 vol% CO, 1 vol% O_2_, 40–60 vol% H_2_, 10% CO_2_, 10% H_2_O and He balance at a GHSV of 60 000 h^−1^).

In the literature,^44^ the CO preferential oxidation reaction was done in the presence of H_2_ were done using gas mixtures comprising of CO, O_2_ and H_2_. Conversely, this feed does not feign a real condition of a reforming system anywhere, besides the stated gases, there is a certain quantity of CO_2_ and H_2_O existent in the feed mixture.^41^ Thus, the effect of CO_2_ and H_2_O addition on the feed composition was studied over the prepared catalysts (Fig. 6). The presence of CO_2_ in the feed gas yields a trivial decrease of the catalytic activity over the reaction temperatures, still, the increase in the conversion of CO over the temperatures is observed. Additionally, the decrease in the formation of CO_2_ from CO was observed at high temperature. The same behaviour has been observed by various authors. As reported in the literature, the presence of CO_2_ acts to disrupt the reaction mechanism due to alteration of the dissemination of the products adsorbed on metal oxide catalysts.^7,45,46^

The present results also showed that the presence of CO_2_ has a superior effect on hydrogen oxidation so that the CO conversion decreases (Fig. 6). However, it has also been reported that the presence of CO_2_ in the feed stream inhibits CO oxidation, due to the formation of carbonates on the metal oxide surface due to the adsorption of CO_2_ which leads to a deactivation of its redox properties of metal oxides. In addition, at high temperatures, the CO_2_ adsorption effect is reduced, which may explain the increase in the catalytic activity over temperature.

**Fig. 7:**
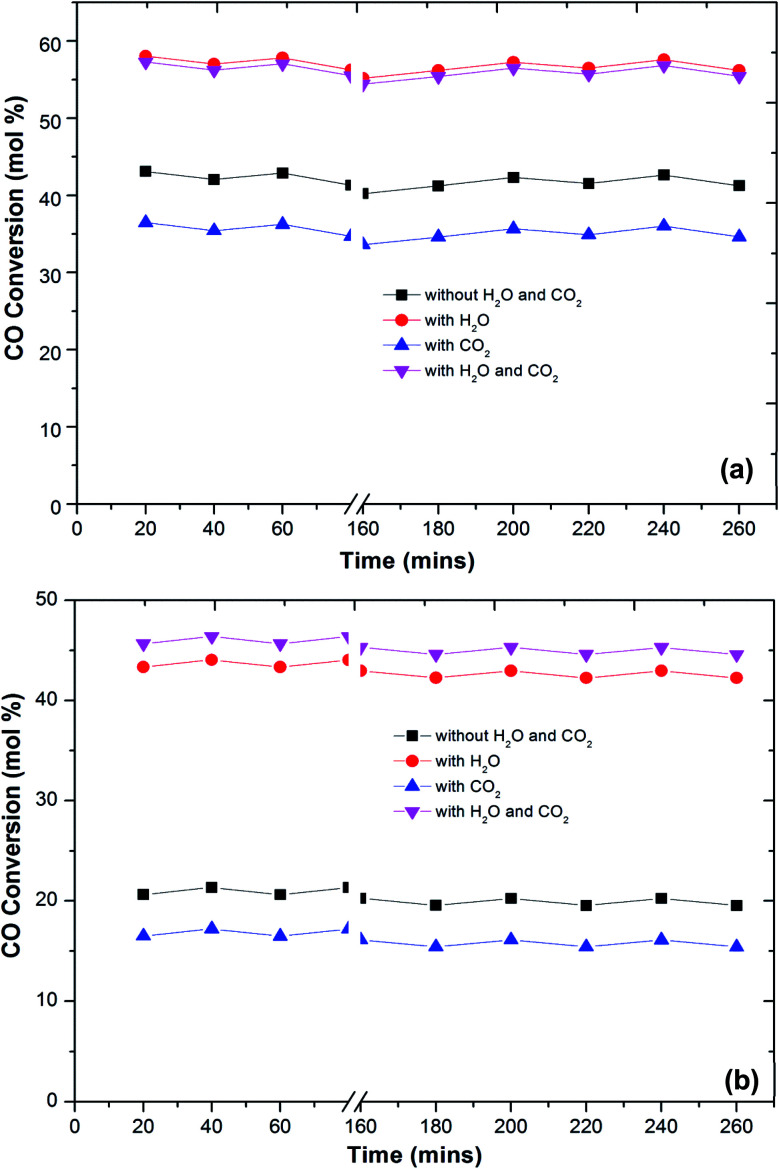
Variation of CO conversion over Cu–Fe/Al_2_O_3_ (a) and Cu/CuFe_2_O_4_ (b) catalyst with reaction temperature of 180 °C at different times (1 vol% CO, 1 vol% O_2_, 60 vol% H_2_, 20% CO_2_, 10% H_2_O and He balance at a GHSV of 60 000 h^−1^).

Water addition to the gas stream affects the catalytic performance of CuFe_2_O_4_ catalysts. This may indicate that the existence of –OH groups on the support surface promotes the catalytic activity. The reformed gas supplied to the PROX reaction after the WGS reaction in real reforming processes contains CO_2_ and H_2_O, which alters the catalyst activity.^8,41,46^ The O_2_ conversion exhibited behaviour similar to the CO conversion and above 120 °C, the O_2_ conversion reached 100%. However, the CO_2_ selectivity decreases by increasing the reaction temperature. The probable reason for the decrease in CO oxidative activity and CO_2_ selectivity with an increase in temperature is the reverse water gas shift reaction. Compared to the results without any addition of CO_2_ or H_2_O, the catalyst activity with an increase in temperature improved with the addition of H_2_O. This result is in agreement with the literature^41,47^ that the addition of H_2_O decreases the activation energy of CO oxidation and H_2_ oxidation such that CO conversion increases significantly without any drastic change in CO_2_ selectivity. This could be due to that the hydroxyl group formed on the catalyst surface by dissociative adsorption of H_2_O acts as a better oxidant than O_2_, thereby increasing the CO and H_2_ oxidation rates and thus the CO conversion (Fig. 7).

One of the most important requirements for PROX catalysts that have to operate in a relatively wide temperature range with good resistance to deactivation caused by H_2_O and CO_2_ in the feed.^41,42,48^ The presence of CO_2_ in the feed will result in the formation of carbonyls or carbonates on the surface of the reaction which further decreases the activity for CO oxidation. Thus, a proper catalyst must be investigated for longer-term stability.

Fig. 7 shows the stability of the catalysts under CO_2_, H_2_O and combined CO_2_ and H_2_O feed. The prepared catalysts showed a stable CO conversion more than 260 min. The same stability is also observed in CO_2_ selectivity. The stability of the catalyst under reaction conditions, in the presence of 10 vol% CO_2_, does not differ from that monitored in the absence of CO_2_, and the CO conversion and selectivity remain constant for more than 260 min. The CO_2_ and H_2_O present in the feed stream of an actual PROX reactor is found to affect the performance of PROX catalysts, for platinum supported catalysts the activation energy for CO oxidation was reduced to 37 kJ from 74 kJ obtained in the absence of H_2_O.^49^ The same trend observed over the present catalysts. The activation energies of CO oxidation under different feed conditions are calculated using the Arrhenius equation. The activation energy for CO oxidation over Cu–Fe/Al_2_O_3_ was 83 kJ which is decreased to 51 kJ in the presence of H_2_O in the feed. This could be due to the H_2_O blocking H_2_ adsorption and allows preferential CO oxidation at higher temperatures where rates are high. The activation energy of CO oxidation was increased to 101 kJ with CO_2_ in the feed and decreased to 59 kJ with a combined H_2_O and CO_2_ feed.

In comparison with other Cu-based catalysts for PROX reaction reported in the literature^19,45,47^ Cu–Fe/Al_2_O_3_ catalyst showed high activity for CO-PROX reaction. For the catalysts reported in the literature, it was found that the strong interaction between the active copper species (probably Cu^+^) and the reducible support (such as Fe_2_O_3_ and CeO_2_) is a prerequisite for the high activity of CO oxidation. The high activity might be due to the small Cu^+^ clusters which were *in situ* formed under hydrothermal conditions due to the chemical interaction with the CuFe_2_O_4_ catalyst.^7,40,46,48^ Moreover, under CO-PROX reaction conditions, Cu was found to exist as Cu^+^. Similar to the CuO systems reported in the literature^45,50–52^ Cu composite systems presented in this work has shown high activity for CO oxidation, and the strong interaction between the two metal components is believed to be responsible for the high activity.

In comparison to the other Cu–Fe based catalysts and Cu based catalysts for PROX reaction, reported in the literature,^15,19,45,47,53,59^ CuFe_2_O_4_ material exhibited high activity (Table 4). These catalysts showed an enhanced performance when compared to the analogues, consisting of Au and Pt (Table 4). There are two kinds of metal species, one interacting with Au or Pt, and likely to form the Au–M or Pt–M alloy in the reduction process, and the other being much more dispersed on alumina and strongly interacting with the supports.^39,55,57,58^ Considering these studies, it was also found that the strong interaction between active copper species (predominantly Cu^+^) and Mn is a prerequisite for a good performance in CO oxidation. Catalytic results suggest that the migration of Cu^2+^ ions into the Mn during the mechanochemical reaction appears to be a thermodynamically favoured, but kinetically limited process.^56^ Forming an active CuFe_2_O_4_ catalyst by the mechanochemical method removes many of the poorly understood variables associated with a process like co-precipitation. A high activity might have therefore arisen on account of small Cu and Mn clusters, which were formed *in situ* under hydrothermal conditions due to the chemical reactions.^40,53,54^ Moreover, under CO PROX process conditions, Cu was found to exist mainly as Cu^+^. Analogously to CuFe_2_O_4_ systems, reported in the literature,^45,50,54,56,59^ Cu–Mn composite catalysts, presented in this work (Table 4), have shown a high relative activity for CO oxidation and a strong interaction between the two metal components, believed to be responsible for the said good performance, stability-wise as well.

**Table 4:** The apparent observed activity of CO conversion (mmol_CO_ s^−1^ kg_cat._^−1^) over Cu based catalysts (temperature of 200 °C)

Catalyst	The rate of CO conversion (mmol_CO_ s^−1^ kg_cat._^−1^)	Reference
Cu–Mn (sol–gel method)	2.93	53
Cu–Mn/Al_2_O_3_	3.32	15
Cu–Ni/Al_2_O_3_	2.91	15
Cu–Mn/ZrO_2_–TiO_2_	2.85	54
Au–Cu/Al_2_O_3_	3.35	55
Cu–Mn commercial catalyst (hopcalite)	3.11	56
Pt–Ni/Al_2_O_3_	3.21	39
Pt–Cu/Al_2_O_3_	3.33	57
Pt–Co/Al_2_O_3_	2.51	58
CuFe_0.4_O_*x*_	1.85	59
CuFe_2_O_4_	2.12	This work
Cu–Fe/Al_2_O_3_	3.96	This work
Cu/CuFe_2_O_4_	4.21	This work
Fe/CuFe_2_O_4_	4.01	This work

## 6 Conclusion

Mössbauer spectroscopy in addition with powder X-ray diffraction has proved to be a very sensitive tool in providing an understanding of the effect of Cu–Fe phase on the selective oxidation of CO with and without H_2_. The Mössbauer data of the Cu and Fe loaded catalyst showed three well-defined sextets belonging to the tetrahedral and octahedral sites of Fe^3+^ ions and confirm that the synthesised materials possess the inverse spinel structure. This indicated that copper combines with iron to form a Cu–Fe supersaturated solid solution. In the CO oxidation (without H_2_), the activities compare as follows at *T*_50_ (the temperatures where 50% CO conversion is achieved): Fe/CuFe_2_O_4_ (120 °C) > CuFe_2_O_4_/Al_2_O_3_ (140 °C) > CuFe_2_O_4_ (140 °C) > Cu/CuFe_2_O_4_. This could be attributed to the reducibility of the iron oxide catalysts in the CuFe_2_O_4_ phase. These results indicate that the introduction of Fe species into CuFe_2_O_4_ catalysts can promote the oxidation of CO. The CO conversion (in the presence of H_2_) and the selectivity to CO_2_ over the other catalysts decrease gradually, indicating that the monometallic phases (*i.e.* CuO and Fe_2_O_3_) are mainly active in hydrogen oxidation. The drop in CO_2_ selectivity, and resulting in lower CO-PROX performance, may be attributed to the generation of an excess of –OH oxidising species.

The activation energy for CO oxidation over Cu–Fe/Al_2_O_3_ was 83 kJ which is decreased to 51 kJ in the presence of H_2_O in the feed. This could be due to the H_2_O blocking H_2_ adsorption and allows preferential CO oxidation at higher temperatures where rates are high. The activation energy of CO oxidation was increased to 101 kJ with CO_2_ in the feed and decreased to 59 kJ with a combined H_2_O and CO_2_ feed. In comparison with the catalyst systems reported in the literature^45,50–52^ Cu composite systems presented in this work has shown high activity for CO oxidation, and the strong interaction between the two metal components is believed to be responsible for the high activity. In addition to their catalytic efficiency, the employed heterogeneous catalysts are inexpensive to produce and do not contain any critical raw materials such as the platinum group metals.

## Conflicts of interest

There are no conflicts to declare.

## Supplementary Material

RA-010-D0RA06969H-s001
