# Supplementary material for: Heterogeneous Cu–Fe oxide catalysts for preferential CO oxidation (PROX) in H2-rich process streams
Source: RSC Adv. 2020 Sep 30;10(59):35792–802. doi: 10.1039/d0ra06969h (PMC9056926; doi:10.1039/d0ra06969h)
Supplement: RA-010-D0RA06969H-s001 [file RA-010-D0RA06969H-s001.pdf]

Electronic Supplementary Material (ESI) for RSC Advances.
This journal is © The Royal Society of Chemistry 2020

Electronic Supplementary Information

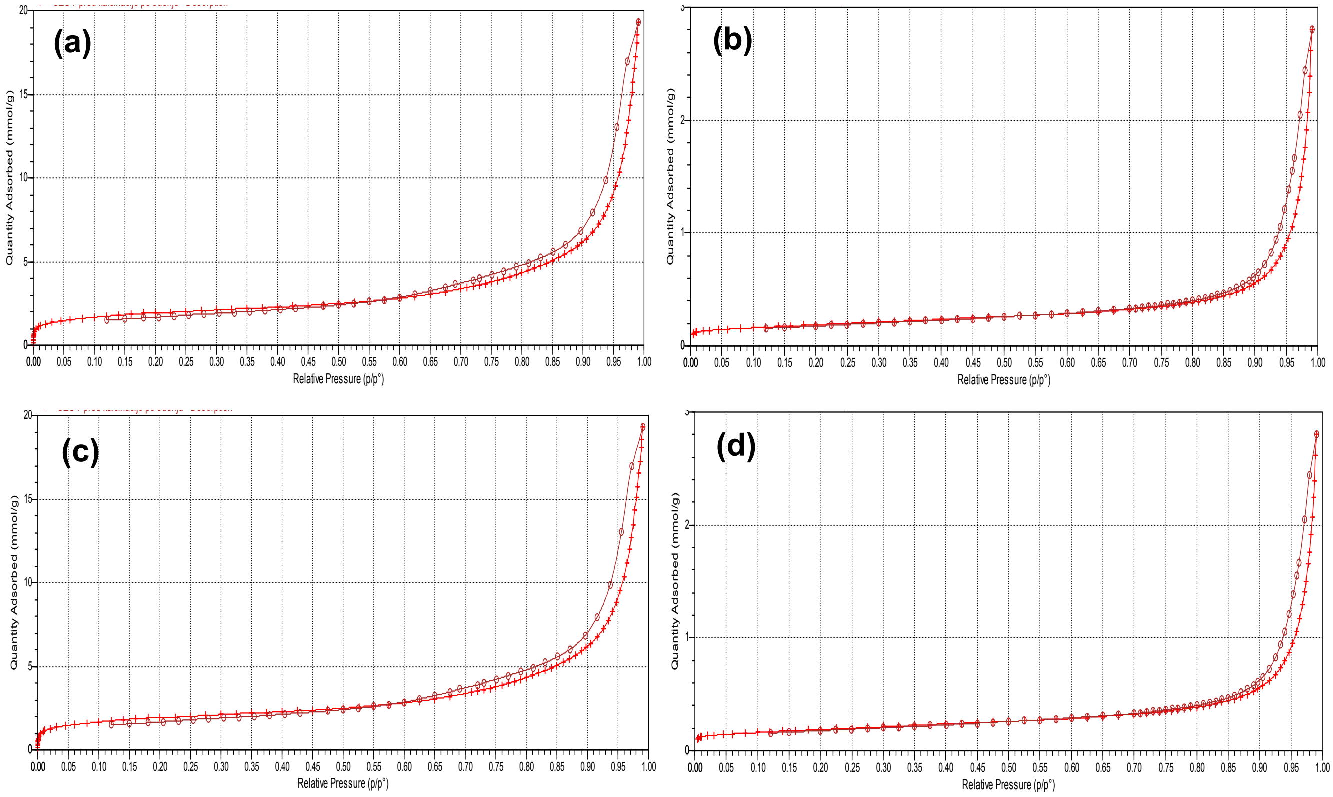

Figure S1: N_2 physisorption isotherms of (a) Cu–Fe/Al_2O_3, (b) $CuFe_2O_4$, (c) Cu/$CuFe_2O_4$ and (d) Fe/$CuFe_2O_4$ catalyst
